# The DNA relaxation activity and covalent complex accumulation of *Mycobacterium tuberculosis *topoisomerase I can be assayed in *Escherichia coli*: application for identification of potential FRET-dye labeling sites

**DOI:** 10.1186/1471-2091-11-41

**Published:** 2010-09-30

**Authors:** Gagandeep Narula, Jennifer Becker, Bokun Cheng, Neil Dani, Maria V Abrenica, Yuk-Ching Tse-Dinh

**Affiliations:** 1 Department of Biochemistry and Molecular Biology, New York Medical College, Valhalla, New York 10595, USA

## Abstract

**Background:**

*Mycobacterium tuberculosis *topoisomerase I (MtTOP1) and *Escherichia coli *topoisomerase I have highly homologous transesterification domains, but the two enzymes have distinctly different C-terminal domains. To investigate the structure-function of MtTOP1 and to target its activity for development of new TB therapy, it is desirable to have a rapid genetic assay for its catalytic activity, and potential bactericidal consequence from accumulation of its covalent complex.

**Results:**

We show that plasmid-encoded recombinant MtTOP1 can complement the temperature sensitive *topA *function of *E. coli *strain AS17. Moreover, expression of MtTOP1-G116 S enzyme with the TOPRIM mutation that inhibits DNA religation results in SOS induction and loss of viability in *E. coli*. The absence of cysteine residues in the MtTOP1 enzyme makes it an attractive system for introduction of potentially informative chemical or spectroscopic probes at specific positions via cysteine mutagenesis. Such probes could be useful for development of high throughput screening (HTS) assays. We employed the AS17 complementation system to screen for sites in MtTOP1 that can tolerate cysteine substitution without loss of complementation function. These cysteine substitution mutants were confirmed to have retained the relaxation activity. One such mutant of MtTOP1 was utilized for fluorescence probe incorporation and fluorescence resonance energy transfer measurement with fluorophore-labeled oligonucleotide substrate.

**Conclusions:**

The DNA relaxation and cleavage complex accumulation of *M. tuberculosis *topoisomerase I can be measured with genetic assays in *E. coli*, facilitating rapid analysis of its activities, and discovery of new TB therapy targeting this essential enzyme.

## Background

DNA topoisomerases maintain the proper topological state of DNA required for vital cellular functions to proceed. The change in DNA topology is catalyzed via concerted breaking and rejoining of DNA coupled with DNA strand passage [1], forming a covalent topoisomerase complex with cleaved DNA as the catalytic intermediate. The accumulation of the covalent complex accounts for the lethal effects of many topoisomerase inhibitors that are utilized as anticancer or antibacterial drugs in therapeutic applications [2-5]. These drugs target type IB and type IIA topoisomerases, classified according to their structural and mechanistic properties. Discovery of similar topoisomerase poison inhibitors for type IA topoisomerases, which include bacterial topoisomerase I enzyme, could lead to the discovery of novel antibacterial agents that can be used for treatment of multi-drug resistant bacterial pathogens. It has been demonstrated via mutants defective in DNA rejoining that accumulation of *E. coli *or *Yersinia pestis *topoisomerase I cleavage complex in *E. coli *leads to rapid bacterial cell death [6,7] validating bacterial topoisomerase I as a new therapeutic target [8]. In addition to poison inhibitors, catalytic inhibitors that act by a different mechanism could also be useful therapeutic agents for bacterial pathogens that require a functional topoisomerase I enzyme for viability or pathogenicity. It has been suggested that at least one type IA topoisomerase enzyme activity must be present in each organism for resolving topological barriers that require single-stranded DNA cleavage [9]. Unlike *E. coli*, which encodes topoisomerase III as a second type IA topoisomerase, *M. tuberculosis *has only one type IA topoisomerase activity. Results from previous transposon mutagenesis studies strongly suggest that the topoisomerase I function is required for *M. tuberculosis *growth and survival during infection [10,11]. *M. tuberculosis *topoisomerase I (MtTOP1) has been proposed as a target for discovery of new TB drugs [12] that are urgently needed due to the large number of human deaths caused by TB globally, and the increasing incidence of TB strains resistant to all available current drugs.

To study the structure-function of MtTOP1 and to monitor its activity, it would be desirable to have a rapid genetic assay for its specific function. The *E. coli *strain AS17 [13] has a mutation in the chromosomal *topA *gene that reduces the topoisomerase I relaxation activity, with growth permissible at 30°C but non-permissive at 42°C unless complemented by recombinant relaxation activity [14,15]. The topoisomerase I enzyme of *E. coli *and *M. tuberculosis *shares a highly homologous transesterification domain (~67,000 kDa) that is conserved among all type IA topoisomerases. It contains the active site tyrosine residue for nucleophilic attack of DNA in the formation of the covalent cleavage complex, and residues in the TOPRIM domain required for coordination of Mg^2+ ^during catalysis [16]. DNA cleavage site selectivity was found to involve the preference of a cytosine at the-4 position relative to the cleavage sites preferred by topoisomerase I and reverse gyrase enzymes in the type IA family of topoisomerases [17-19]. However, the transesterification domain cannot carry out relaxation of negative supercoils in the absence of the C-terminal domain [14]. The C-terminal domains of *E. coli *and *M. tuberculosis *topoisomerase I belong to two distinct families. The C-terminal domain of *E. coli *topoisomerase I has multiple tetracysteine motifs for coordination of Zn(II) ions required for relaxation activity [20], while the C-terminal domains of topoisomerase I found in *Mycobacterium smegmatis *and *M. tuberculosis *were found not to have any Zn(II) coordinating tetracysteine motifs [21].

In addition to the difference between the C-terminal domain sequences of *Mycobacterium *and *E. coli *topoisomerase I, it is also unclear if MtTOP1 can be functional in *E. coli *due to the significant difference in codon usage between *E. coli *and *M. tuberculosis*. The structure-function study of MtTOP1 would be greatly facilitated if a rapid genetic assay system utilizing *E. coli *can be established. For further study of the enzymatic properties of MtTOP1, we have initiated biochemical studies of MtTOP1 to take advantage of the absence of cysteine residue in its sequence. If a unique cysteine can be introduced into a specific position via site-directed mutagenesis without diminishing the catalytic activity, spectroscopic or chemical probes can then be introduced to study the protein-DNA interactions involved in catalysis. Spectrophotometric analysis utilizing these probes could potentially be developed into HTS assay for identification and characterization of small molecule inhibitors of MtTOP1.

In this study, we demonstrated that plasmid encoding MtTOP1 can complement *E. coli *AS17 with temperature sensitive *topA *[13] for growth at 42°C. In addition to demonstrating that the relaxation activity of wild-type MtTOP1 was functional in *E. coli*, we also showed that when a mutant version of MtTOP1, with the G116 S TOPRIM mutation that inhibits DNA religation [6] was overexpressed in *E. coli*, the SOS response is induced along with a significant decrease in viability. Therefore, the accumulation of cleavage complex formed by MtTOP1 can also be assayed in *E. coli*. We utilized the *E. coli *genetic system to identify a number of positions in MtTOP1 that could be mutated to cysteine with little or no loss of relaxation activity. The retention of relaxation activity was confirmed after the expression and purification of the mutant enzymes. One such cysteine substitution mutant enzyme MtTOP1-K524C was conjugated to a fluorescence dye for fluorescence resonance energy transfer (FRET) study with fluorescence-labeled oligonucleotide substrates.

## Results

### Complementation of *topA *function in *E. coli *AS17 for growth at 42°C by *M. tuberculosis *topoisomerase I

**Figure 1:**
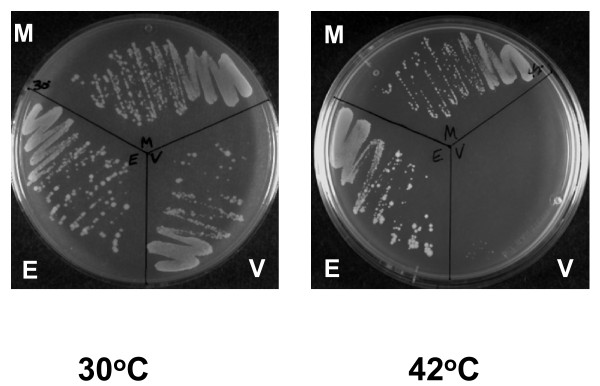
**Complementation of *topA^ts ^*function in *E. coli *AS17 by recombinant *M. tuberculosis *topoisomerase I**. Transformants of *E. coli *AS17 with pMTOP (M), pETOP (E) and vector pBAD/thio (V) were streaked on LB plates with 100 μg/ml ampicillin and incubated at the indicated temperature for 48 h.

*E. coli *AS17 transformed with plasmid pMTOP was streaked onto LB plates with ampicillin and incubated at either 30°C or 42°C. Plasmid pETOP expressing recombinant *E. coli *topoisomerase I in the same vector was used as positive control. Complementation for growth at 42°C could be observed with both pMTOP and pETOP. No complementation for growth at 42°C could be seen with the vector pBAD/thio (Figure 1). This result showed that the recombinant MtTOP1 interacts with the chromosomal DNA in *E. coli*, and the background uninduced expression of MtTOP1 from the BAD promoter in pMTOP clone was sufficient for complementation of the temperature sensitive *topA *function in *E. coli *AS17 for growth at 42°C. Even though *E. coli *and *M. tuberculosis *topoisomerase I enzymes have different C-terminal domain sequences, the conservation of the N-terminal transesterification domain in MtTOP1 is likely to allow MtTOP1 to bind to single-stranded DNA associated with negatively supercoiled region of the *E. coli *chromosome to remove excess negative supercoils.

### Cell killing from accumulation of mutant MtTOP1 covalent complex in *E. coli*

The TOPRIM motif DxDxxG is strictly conserved in type IA topoisomerase sequences and plays an essential role for divalent ion coordination and cleavage-religation of DNA during catalysis [16,22,23]. For recombinant *E. coli *or *Y. pestis *topoisomerase I, mutation of the first aspartate residue in the TOPRIM motif to asparagine has been shown to be extremely lethal to *E. coli *[7]. Substitution of the conserved glycine in the TOPRIM motif with serine in *E. coli *or *Y. pestis *topoisomerase I resulted in mutant topoisomerase I enzyme capable of DNA cleavage but defective in DNA religation [6]. Induction of the BAD promoter regulating the expression of these Gly to Ser mutant *E. coli *and *Y. pestis *topoisomerases resulted in loss of viability of the host *E. coli *cells [6].

An attempt was made to introduce the Asn substitution at the first Asp of the TOPRIM motif found in MtTOP1 (Asp111) by site-directed mutagenesis. Clones of pMTOP with the desired D111N mutation as a single substitution could not be isolated after transformation of commonly used *E. coli *cloning strains even in the presence of 2% glucose to suppress transcription from the BAD promoter regardless of the *recA *genotype of the *E. coli *host cells. This was likely due to the toxic effect of the mutant MtTOP1 background expression on *E. coli *viability. All the isolated clones had second site mutations, similar to what was observed during attempted construction of *E. coli *and *Y. pestis *topoisomerase I mutants with the D111N mutation [7]. This suggested that *E. coli *host cells can be very vulnerable to accumulation of covalent cleavage complex formed by recombinant MtTOP1 enzyme *in vivo*.

**Figure 2:**
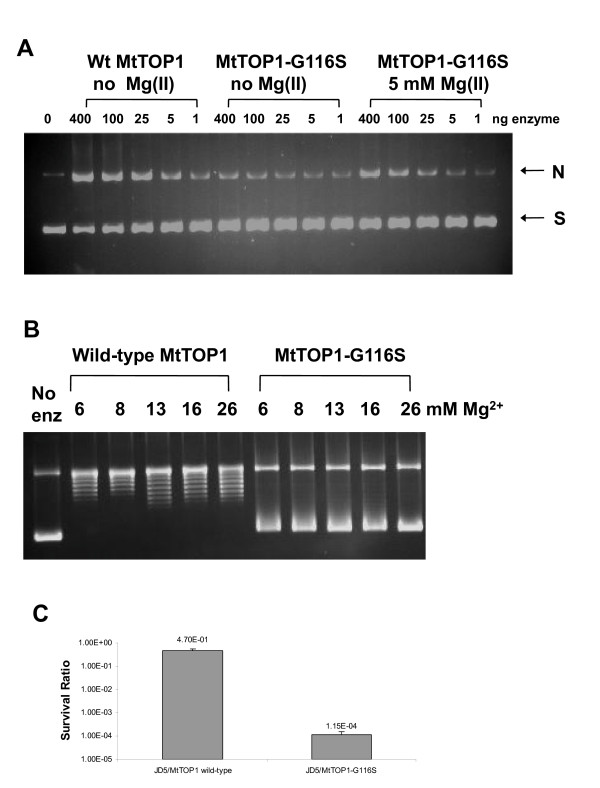
**Analysis of biochemical properties of MtTOP1-G116 S enzyme and the lethal consequence of its overexpression**. **(A) **Magnesium dependence of DNA cleavage. Wild-type and G116 S MtTOP1 proteins were assayed for cleavage of negatively supercoiled plasmid DNA with agarose gel electrophoresis in the presence of 0.5 μg/ml ethidium bromide. N: nicked DNA; S: supercoiled DNA **(B) **MtTOP1-G116 S mutant has no relaxation activity even in the presence of excess Mg(II) ions. Wild-type and G116 S mutant MtTOP1 (400 ng) were assayed in a 20 μl reaction with 10 mM Tris-HCl, pH 8.0, 50 mM NaCl, 0.1 mg/ml gelatin and the indicated concentration of MgCl_2 _at 37°C for 30 min. Agarose gel electrophoresis in the absence of ethidium bromide was used to separate the supercoiled DNA from the slower migrating relaxed topoisomers. **(C) **Loss of viability of *E. coli *JD5 after induction of MtTOP1-G116 S expression with arabinose. Viable colony counts determined 2 h after addition of 0.2% arabinose were divided by viable colony counts from control non-induced cultures to obtain the survival ratio.

The G116 S mutation in *E. coli *topoisomerase I has a lesser lethal effect when compared to the D111N mutation because it accumulates lower amounts of cleavage complex [7]. The Ser substitution was successfully made at Gly116 of the MtTOP1 TOPRIM motif in pMTOP and a clone with no second-site mutation was isolated in the presence of 2% glucose. The mutant MtTOP1-G116 S enzyme was expressed by arabinose induction and purified. Mg^2+ ^ions are required for DNA religation and relaxation activity of all type IA topoisomerases [24,25]. Mg^2+ ^ions did not have to be added for DNA cleavage to be observed by wild-type MtTOP1 (Figure 2A), but the DNA cleavage activity of MtTOP1-G116 S is Mg^2+ ^dependent, similar to the Gly to Ser substitution mutants previously characterized for *E. coli *and *Y. pestis *topoisomerase I [6]. Quantitation of the nicked DNA formed (additional file 1: Quantitation of DNA nicking by MtTOP1-G116S) showed that ~30% of the input DNA could be converted to the nicked form by MtTOP1-G116 S in the presence of MgCl_2_. No relaxation activity could be detected for MtTOP1-G116 S as expected from the loss of DNA religation activity even in the presence of high concentrations of Mg^2+ ^ions (Figure 2B).

Plasmid pMTOP-G116S was transformed into *E. coli *strain JD5 and the transformants were first isolated in the presence of 2% glucose. The transformants were then replica plated onto Xgal indicator plate with 0.002% arabinose to increase the level of synthesis of mutant MtTOP1-G116 S. SOS response to the accumulated cleavage complex was evident from the blue color of the colonies in the presence of 0.002% arabinose resulting from induction of the *lacZ *gene under the control of the *dinD1 *promoter in *E. coli *JD5 [6]. Treatment of an exponential phase culture of JD5/pMTOP-G116 S with 0.2% arabinose for 2 h resulted in a viable colony count ratio of ~10^-4 ^when the number of viable colonies from induced culture were divided by the viable colony counts from the non-induced culture (Figure 2C). Induction of wild-type MtTOP1 by 0.2% arabinose reduced the viable counts of *E. coli *JD5 by only ~2 fold. The degree of loss of viability from the Gly to Ser mutation in pMTOP was similar to that observed previously for the same mutation in pETOP [6]. These results showed that the accumulation of the covalent cleavage complex formed by recombinant MtTOP1 in *E. coli *could result in SOS induction and bacterial cell death.

### Assay of relaxation activity of cysteine-substitution mutants of MtTOP1 by complementation of *E. coli topA *function

**Figure 3:**
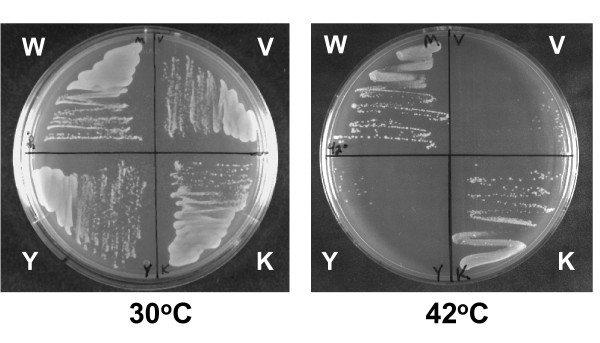
**Complementation assay of *E. coli *AS17 by pLIC-MTOP**. encoding wild-type MtTOP1 (W), and mutants MtTOP1-Y342A (Y) and MtTOP1-K524C (K). V: LIC vector

**Figure 4:**
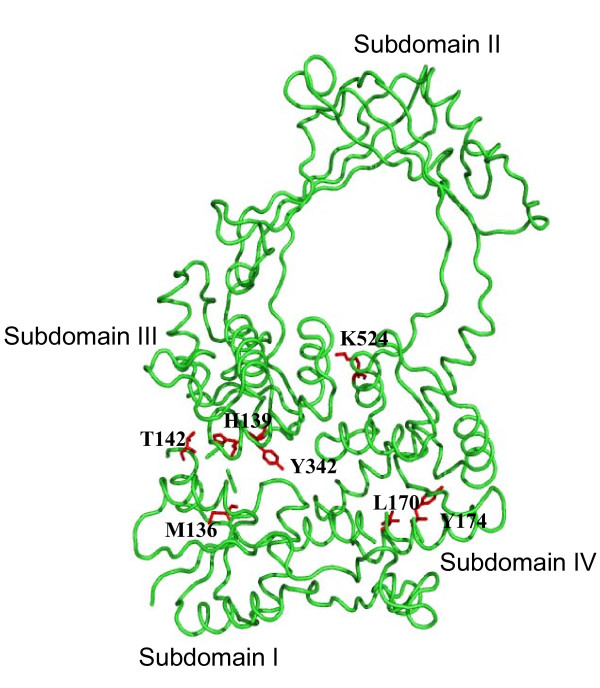
**Sites of cysteine substitutions shown on structural model of MtTOP1 N-terminal transesterification domain**. The active site tyrosine Y342 is also shown for reference.

MtTOP1 protein from *M. tuberculosis *H37Rv has the very useful property of having no cysteine residues in its sequence. It is thus an ideal model system for biochemical studies where a unique cysteine can be introduced by site-directed mutagenesis to facilitate chemical conjugation of fluorescence or affinity probe at a specific position. Expression and purification procedures utilizing plasmid pLIC-MTOP have been developed to produce milligram quantities of soluble and active MtTOP1 protein [17]. Even though the pLIC-MTOP plasmid requires induction of the T7 promoter for high level protein expression, there is likely to be a certain level of constitutive background expression in the absence of T7 RNA polymerase induction, similar to background expression of MtTOP1 from pMTOP in the absence of arabinose induction of the BAD promoter. This background expression in pLIC-MTOP was found to be sufficient for complementation of the *topA *function of *E. coli *AS17 for growth when plates incubated at 30°C and 42°C were compared (Figure 3). Transformation of *E. coli *AS17 with the corresponding vector or plasmid expressing the active site mutant MtTOP1-Y342A did not result in complementation. A number of residues in MtTOP1 were selected for cysteine substitution based on the criteria that the residues are not highly conserved in identity, but are positioned near strictly conserved residues [26]. These are shown on the model structure of MtTOP1 in MODBASE [27] derived from the crystal structure of the N-terminal domain of *E. coli *topoisomerase I 1MW9 (Figure 4). pLIC-MTOP derivatives with these single cysteine substitution were transformed into *E. coli *AS17. Complementation of the temperature sensitive *topA *function in *E. coli *AS17 was assayed by growth of the transformants at 42°C, as shown for MtTOP1-K524C mutant (Figure 3), or more quantitatively by plating serial dilutions of the AS17 transformant culture and comparing the number of viable colonies obtained after incubation at either 30°C or 42°C (Table 1). There was no complementation from the Y342A or G116 S MtTOP1 mutants as expected. The results from the cysteine substitutions mutants suggested that cysteine substitution at most of the positions tested could be tolerated without significantly affecting the ability of MtTOP1 to complement the *topA *chromosomal mutation in AS17 for growth at the non-permissive temperature.

**Table 1:** Effect of MtTOP1 substitutions on the complementation of *topA*^*ts *^function in *E. coli *AS17 at the non-permissive temperature

Plasmid	Complementation ratio
pLIC vector	3.9 × 10^-5 ^± 3.3 × 10^-5^

pLIC-ETOP	0.58 ± 0.21

pLIC-MTOP	0.53 ± 0.24

pLIC-MTOP-Y342A	1.1 × 10^-4 ^± 1.1 × 10^-4^

pLIC-MTOP-G116S	3.3 × 10^-5 ^± 2.0 × 10^-5^

pLIC-MTOP-M136C	2.7 × 10^-4 ^± 4.1 × 10^-4^

pLIC-MTOP-H139C	0.19 ± 0.15

pLIC-MTOP-T142C	0.097 ± 0.029

pLIC-MTOP-L170C	0.32 ± 0.16

pLIC-MTOP-Y174C	0.18 ± 0.06

pLIC-MTOP-K524C	0.33 ± 0.17

Serial dilutions of overnight cultures of *E. coli *AS17 transformants were plated on LB plates with kanamycin and incubated for 2 days at either 30°C or 42°C. Colony counts from 42°C incubation were divided by colony counts from 30°C to obtain the complementation ratios. The results represent the average and standard deviation from at least three measurements.

**Figure 5:**
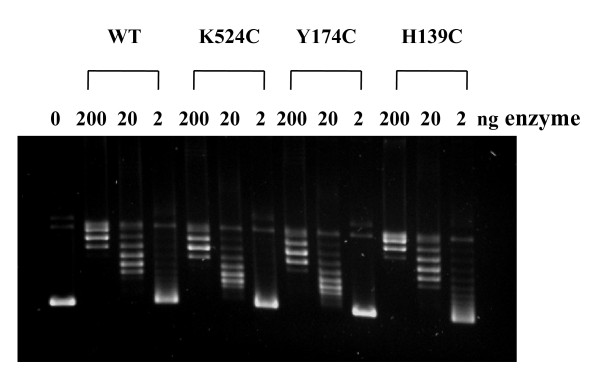
**Relaxation activity assay of H139C, Y174C and K524C mutants of MtTOP1**. Serial dilutions of purified wild-type MtTOP1 and mutant derivatives with cysteine substitutions at H139, Y174 and K524 were added to 20 μL reaction volume of 10 mM Tris-HCl, pH 8.0, 50 mM NaCl, 0.1 mg/mL gelatin, 6 mM MgCl_2 _and 0.2 μg of supercoiled plasmid DNA. Incubation was at 37°C for 30 min.

To confirm the retention of relaxation activity after the cysteine substitution, mutant MtTOP1 enzymes with cysteine substitutions at H139, Y174, and K524 residues located in different regions of the enzyme were expressed and purified for further characterization. In vitro assay of the relaxation activity (Figure 5) confirmed that these enzymes with a single cysteine introduced by mutagenesis had close to wild-type relaxation activity.

### CPM-MtTOP1-K524C relaxation activity and FRET experiments

**Figure 6:**
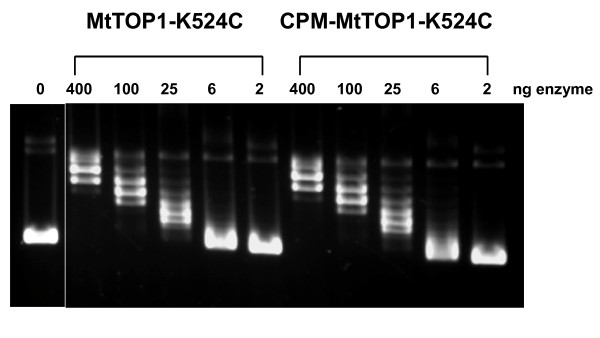
**Relaxation activity of CPM labeled MtTOP1-K524C mutant**. Serial dilutions of unlabeled MtTOP1-K524C mutant and CPM labeled MtTOP1-K524C mutant proteins were assayed for relaxation of supercoiled plasmid DNA.

**Figure 7:**
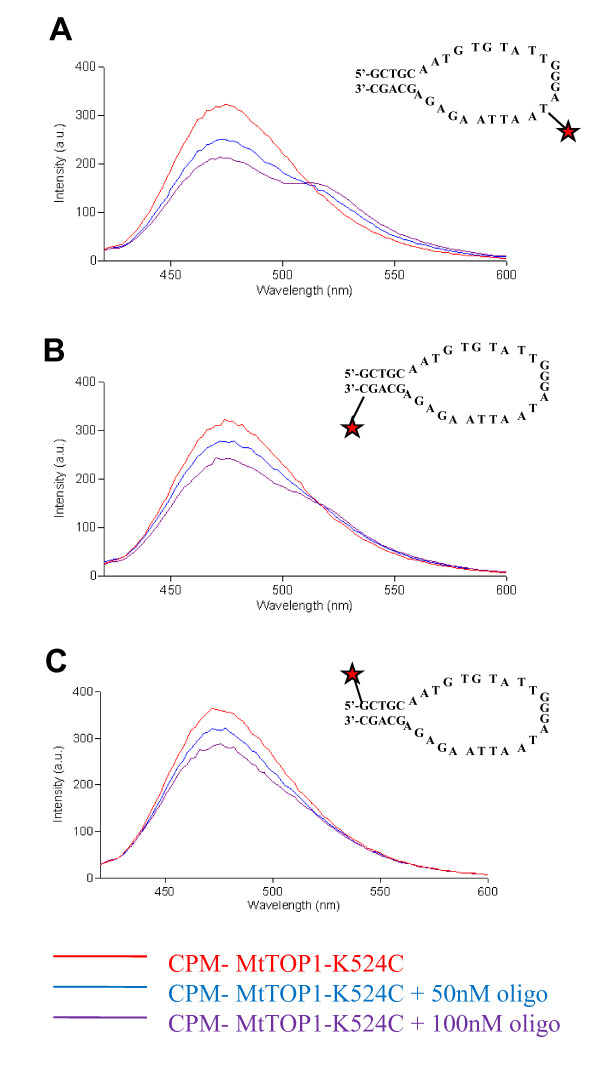
**FRET between CPM-MtTOP1-K524C and fluorescein labeled oligonucleotides**. Increase in fluorescein fluorescence (520 nm) was observed when CPM-MtTOP1-K524C was titrated with increasing concentrations of oligo internally labeled with fluorescein indicating the occurrence of FRET **(A)**. Little or no increase in fluorescein fluorescence was observed when titrations were carried out with oligo labeled at the 3' end **(B) **or the 5' end **(C)**.

MtTOP1-K524C was labeled with CPM as described in Materials and Methods. After labeling, the activity of the labeled enzyme was checked by relaxation activity assay. As shown in Figure 6, labeling with CPM did not affect the activity of the K524C mutant enzyme. The labeled MtTOP1-K524C demonstrated relaxation activity comparable to unlabeled MtTOP1-K524C. For the FRET experiments, a 35-base oligonucleotide substrate was designed to have a stem-loop structure providing the enzyme with both a single-stranded and a double-stranded region for potential interaction. This oligo was labeled with fluorescein at 3 positions: 3' end, 5' end and an internal location within the single-stranded loop. Upon titration of CPM-MtTOP1-K524C with substrate labeled with fluorescein at either the 5' or 3' end, no FRET was observed (Figure 7B, C). However, upon titration with substrate labeled with fluorescein in the single-stranded loop, a large drop in the CPM emission (470 nm) and a corresponding increase in the fluorescein emission (520 nm) indicated the occurrence of FRET (Figure 7A). The results obtained from the FRET experiments were used to calculate the energy transfer efficiency (*E*). The maximum transfer efficiency was observed upon titration with the internally labeled oligo (0.72). Titration with the 3' and the 5' labeled oligo demonstrated transfer efficiencies of 0.45 and 0.39 respectively.

We found that MtTOP1-H139C protein could also be labeled with fluorophores with no significant loss of relaxation activity (results not shown). However, FRET was not observed in similar studies of labeled MtTOP1-H139C with oligonucleotide substrates.

## Discussion

Due to the requirement of topoisomerase I function for *M. tuberculosis *growth and survival during infection as indicated by transposon mutagenesis studies [10,11], discovery of specific inhibitors of MtTOP1 could lead to development of novel therapeutics urgently needed for treatment of multi-drug resistant TB. The utilization of MtTOP1 as a potential new drug target would be facilitated by development of simple assays for the catalytic function of the enzyme. It is also desirable to identify poison inhibitors that can result in accumulation of the covalent complex of MtTOP1, since such topoisomerase poison inhibitors are expected to be bactericidal, and might be useful when used in combination therapy with current TB drugs such as fluoroquinolones [28].

The results described here demonstrated that inhibition of DNA religation by recombinant MtTOP1 would result in killing of the *E. coli *host cells. Increased expression of recombinant MtTOP1 should result in hypersensitivity to its poison inhibitors. The catalytic function of recombinant MtTOP1 could also be assessed easily from its complementation of the temperature sensitive *topA *function in *E. coli *AS17 at the non-permissive temperature. Despite a potential problem from difference in codon preference of the different organisms, and divergence of the topoisomerase I C-terminal domain sequences, recombinant MtTOP1 could still form covalent cleavage complex on the *E. coli *chromosome and remove excess negative supercoils so that viable functions can proceed.

The *E. coli *cell based assays are useful in structure-function studies of the enzyme. Mutations that abolish the catalytic activity after amino acid substitutions, such as Y342A and G116 S, could be identified readily. In another approach to study the enzyme mechanism, cysteine mutagenesis sites in different regions of the enzyme which do not affect the enzyme activity could also be determined rapidly by using the *E. coli *AS17 complementation assay. These cysteine substitutions are designed to take advantage of the absence of cysteine in wild-type MtTOP1 sequence to introduce a sulfhydryl group that would allow conjugation of a molecular probe at a unique position on the enzyme. Cysteine mutants that could complement AS17 for growth at non-permissive temperature were found to have close to wild-type relaxation activity when purified, confirming the effectiveness of the genetic assay.

For CPM fluorophore introduced at residue K524, FRET was observed with fluorescein fluorophore placed in the single stranded region of the substrate, but not at the double stranded region. In the mechanistic model proposed for the relaxation of DNA by topoisomerase I [25], a single strand of DNA passes into the interior cavity of the enzyme in order to change the linking number of the DNA substrate. That would place the loop region of the single-stranded DNA in the vicinity of K524, accounting for FRET between CPM-MtTOP1-K524C and fluorescein in the single-stranded loop region of the substrate. The double-stranded region of the DNA substrate may extend from the DNA binding groove formed by subdomains I and IV [29], further away from the interior cavity of the enzyme and K524, explaining the absence of FRET when fluorescein was placed at the 5' or 3' end of the DNA substrate. Although H139 is closer to the active site tyrosine Y342 than K524, binding of the stem region of the DNA substrate in subdomain IV would place the DNA in opposite direction from the active site away from H139, too far for FRET to take place. We tried to attach fluorescent probes with the Y174C mutant, but the labeling efficiency was poor. Other cysteine substitution mutants will be explored in future related mechanistic studies. Cysteine substitution mutants of MtTOP1 could potentially be utilized for development of enzyme based HTS assays for inhibitors of MtTOP1 activity.

The E. coli cell based assays could also be useful for testing potential MtTOP1 inhibitors. Whole cell screenings has identified a large number of small molecules that can inhibit the growth of *M. tuberculosis *[30,31], but it is often difficult to quickly determine the cellular target of antibacterial compounds without extensive follow up investigations. Previous studies showed that the *topA *function in *E. coli *AS17 can also be complemented by the type IB topoisomerase gene from *Saccharomyces cerevisae *[32]. Since the type IA and type IB topoisomerases have distinctly different structures and mechanisms, an inhibitor with high specificity against type IA topoisomerase would affect growth of *E. coli *AS17 complemented by MtTOP1 activity at the non-permissive temperature, but should have much lower antibacterial activity against *E. coli *AS17 complemented by yeast topoisomerase I. This complementation assay system should facilitate the identification of lead compounds that have antibacterial activity due to interactions with *M. tuberculosis *topoisomerase I in their mechanism of action. Toxic compounds that reduce cell viability by interaction with cell membranes or DNA can be eliminated quickly in the pursuit of useful drug leads. To facilitate the screening, permeability of *E. coli *AS17 to small molecules can be enhanced by incorporation of mutations that affect the drug transporters [33] or membrane structure [34].

## Conclusions

Rapid genetic assays in *E. coli *have been described here for assessing the relaxation activity and accumulation of covalent cleavage complex of recombinant MtTOP1. The genetic assay was used to identify positions in MtTOP1 that can be converted to cysteine residues without loss of function. One such cysteine was utilized as FRET-dye labeling sites to monitor interaction with DNA substrate.

## Methods

### MtTOP1 clones

PCR reaction with *PfuUltra *II Fusion HS DNA polymerase (from Stratagene) and genomic DNA of *M. tuberculosis *strain H37Rv as template was used to generate the coding sequence of MtTOP1 (gene Rv3646c). The PCR product was cloned into plasmid pBAD/thio using the TOPO cloning kit from Invitrogen to generate plasmid pBAD/thioMTOP. The 5'-PCR primer (5'AACCATGGCTGACCCGAAAACGAAGGGCCG-3') contained a recognition site for the restriction enzyme *Nco *I, which was used to remove the sequence coding for thioredoxin fusion tag from plasmid pBAD/thioMTOP. Following ligation and transformation of the restriction product, plasmid pMTOP which has MtTOP1 expressed under the control of the BAD promoter was obtained. Plamid pLIC-MTOP with expression of MtTOP1 under the control of the T7 promoter has been described previously [17]. Site directed mutagenesis to introduce single amino acid substitutions were carried out with QuikChange procedures using the *PfuUltra *II Fusion HS DNA polymerase. Topoisomerase gene coding regions in all isolated plasmid clones were checked by DNA sequencing to confirm that no other mutations were present.

### Complementation of *topA *function in *E. coli *AS17 by recombinant topoisomerase plasmid clones

*E. coli *AS17 (F^- ^*topA17*(*am*) pLL1(*Tet supD43,74*)) [13] transformants were grown overnight at 30°C, in Luria Bertani (LB) broth containing tetracycline (15 μg/ml) and antibiotics for selection of plasmid clones. Overnight cultures were serially diluted in sterile phosphate buffered saline and each dilution was plated on four LB agar plates with antibiotics for selection of plasmid. Two of the plates were incubated at 30°C, while the other two plates were incubated at 42°C. The viable colonies were counted after 48 hours of incubation at the respective temperatures to determine colony counts in cfu/ml. The entire study was repeated three times minimum for each plasmid clone. Complementation ratios were calculated by dividing the cfu/ml at 42°C by the cfu/ml at 30°C for each individual plasmid clone.

### SOS induction and cell killing assays

Plasmid pMTOP expressing wild-type or mutant MtTOP1-G116 S proteins under the control of the BAD promoter were transformed into *E. coli *JD5 [JM103 *dinDl *::MudI1734(KmR *lacZ*)] with β-galactosidase gene under the control of the SOS regulon [6]. Transformants isolated from LB plates with 100 μg/ml ampicillin and 2% glucose were replicated onto LB plates with 35 μg/ml X-gal, 100 μg/ml ampicillin and 0.002% arabinose. SOS induction was indicated by the appearance of blue colonies after overnight incubation at 37°C as previously described for the isolation of SOS-inducing mutants of *Y. pestis *topoisomerase I [6]. To measure the effect of mutant MtTOP1 expression on viability, early exponential phase culture (OD_600 _= 0.4) of *E. coli *JD5 transformants in LB medium with ampicillin (100 μg/ml) was induced with 0.2% arabinose for 2 h before serial dilution and plating on LB plates with 2% glucose and ampicillin, followed by overnight incubation at 37°C. The number of colonies (cfu/ml) obtained from the induced culture was divided by the number of colonies from the control non-induced culture to obtain the survival ratio.

### Topoisomerase activity assay

MtTOP1-G116 S mutant enzyme expressed by induction of the BAD promoter with 0.2% arabinose in *E. coli *strain GP200, a *topA *deletion mutant [35] was purified as described previously [36]. Recombinant MtTOP1 proteins with single-cysteine substitutions were expressed via induction of the T7 promoter in *E. coli *ArcticExpress(DE3) RP strain (from Stratagene) and purified to homogeneity with the same procedures used for wild-type MtTOP1 [17]. The relaxation activity assay was carried out with negatively supercoiled plasmid DNA substrate as described [17]. The cleavage of supercoiled plasmid DNA by MtTOP1 was assayed according to published procedures [6].

### CPM (7-diethylamino-3-(4'-maleimidylphenyl)-4-methylcoumarin) labeling of MtTOP1-K524C protein

In preparation of the labeling reaction, a 32 μM solution of MtTOP1-K524C in storage buffer (100 mM potassium phosphate pH 7.4, 0.2 mM EDTA, 50% glycerol) was dialyzed against labeling buffer (100 mM potassium phosphate, pH 7.4, 50 mM KCl, 10% glycerol) overnight at 4°C. After dialysis, the protein concentration was determined to be 16 μM. The protein solution was incubated with 100 fold excess of TCEP (tris(2-carboxyethyl)phosphine) at room temperature for 10 min to keep the cysteine residue in the reduced state. Labeling was carried out by addition of 20 fold excess of CPM (Molecular Probes). Before and after each addition, the tube was flushed with 95% nitrogen gas. After CPM addition, the labeling reaction was allowed to proceed on ice for 6 hours. Removal of free dye was achieved by dialysis against labeling buffer. The labeled protein was analyzed by SDS-polyacrylamide gel electrophoresis and visualized by fluorescence from the incorporated CPM with the Storm 860 PhosphorImager and subsequently staining with MicrowaveBlue (from Protiga).

### FRET experiments

FRET experiments were performed in SCAN using the Varian Cary Eclipse fluorescence spectrophotometer. Excitation wavelength was set at 394 nm and emission was measured between 400-600 nm. The excitation and emission slits were set at 5 and 10 nm respectively. All FRET experiments were performed in labeling buffer with 1 mM MgCl_2 _at room temperature. CPM labeled MtTOP1-K524C (750 nM) was titrated with 50 and 100 nM solutions of oligonucleotide substrate (5'-GCTGCAATGTGATTTGGGATAATTAAGAGAGCAGC-3') modified with fluorescein at the 5' or 3' end (supplied by Sigma Genosys), or fluorescein attached via a 6 carbon linker to position 5 of the thymine base at the 20^th ^nucleotide (custom synthesized by Integrated DNA Technologies). Control readings for the donor spectrum were performed by titrating MtTOP1-CPM K524C with buffer. Control readings for the acceptor spectrum were performed at excitation wavelength 495 nm by titrating MtTOP1-CPM K524C with 50 and 100 nM of fluorescein labeled oligos.

### FRET efficiency calculations

FRET efficiency calculations were performed as described [37]. The control readings for the donor spectrum were subtracted from the FRET readings and the values obtained were divided by the control readings for the acceptor spectrum to give (ratio) _A _using the following equation:

$${\left(ratio\right)}_{A}=(F_{DA}^{\lambda D}-a\cdot F_{D}^{\lambda D})/F_{DA}^{\lambda D}$$

Since (ratio)_A _is linearly dependent on the energy transfer efficiency (*E*), *E *was calculated using the following equation,

$$E=\{(ratio_{A})-(\varepsilon_{A}^{\lambda D}/\varepsilon_{A}^{\lambda A})\}/\{d^{+}\cdot (\varepsilon_{D}^{\lambda D}/\varepsilon_{A}^{\lambda A})\}$$

where d^+ ^= fraction of donor labeled molecules.

## Authors' contributions

GN carried out the FRET measurements. JB characterized the complementation of *E. coli *AS17 by MtTOP1 and its mutant derivatives. GN and ND purified and assayed the cysteine substitution mutants of MtTOP1. BC purified the MtTOP1-G116 S mutant enzyme and carried out the relaxation and DNA cleavage assays. MVA measured the cell killing by MtTOP1-G116 S expression. YT conceived the study, and participated in the design and coordination and drafted the manuscript. All authors read and approved the final manuscript.

## Supplementary Material

Additional file 1 **Quantitation of DNA nicking by MtTOP1-G116 S.** The percent of nicked DNA in each lane of Figure 2A was quantitated by densitometry analysis. The increase in percent nicked DNA relative to the control lane with no enzyme present is shown here. Click here for file
