# Supplementary material for: The DNA relaxation activity and covalent complex accumulation of Mycobacterium tuberculosis topoisomerase I can be assayed in Escherichia coli: application for identification of potential FRET-dye labeling sites
Source: BMC Biochem. 2010 Sep 30;11:41. doi: 10.1186/1471-2091-11-41 (PMC2958883; doi:10.1186/1471-2091-11-41)

Additional File 1: Quantitation of DNA nicking by MtTOP1-G116S

The percent of nicked DNA in each lane of Figure 2A was quantitated by densitometry analysis. The increase in percent nicked DNA relative to the control lane with no enzyme present is shown here.

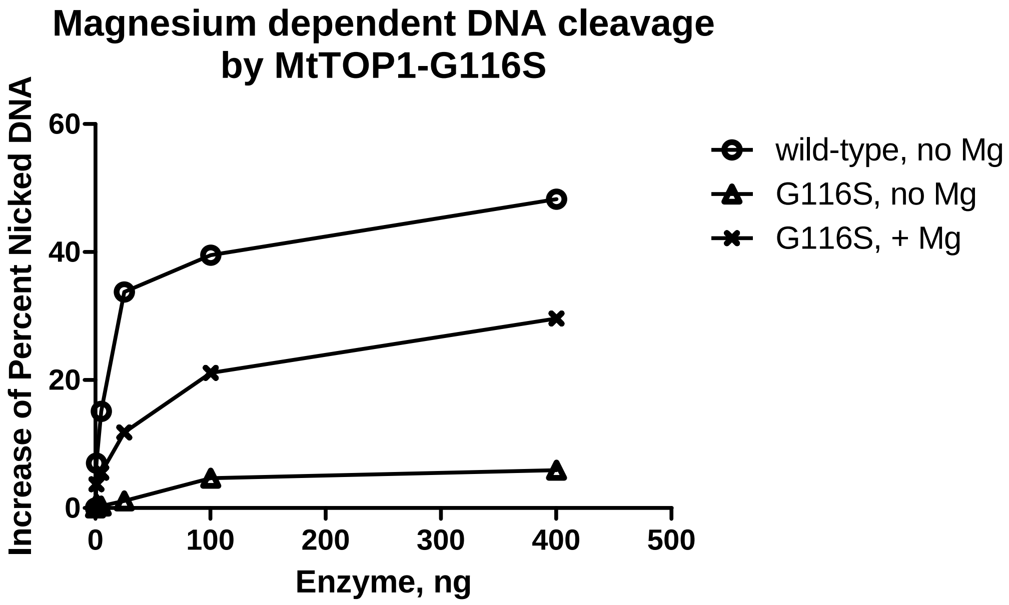

Supplement: Additional file 1 — Quantitation of DNA nicking by MtTOP1-G116 S. The percent of nicked DNA in each lane of Figure 2A was quantitated by densitometry analysis. The increase in percent nicked DNA relative to the control lane with no enzyme present is shown here. [file 1471-2091-11-41-S1.PDF]
